# The Role of the Vagus Nerve in Cancer Prognosis: A Systematic and a Comprehensive Review

**DOI:** 10.1155/2018/1236787

**Published:** 2018-07-02

**Authors:** Marijke De Couck, Ralf Caers, David Spiegel, Yori Gidron

**Affiliations:** ^1^Mental Health and Wellbeing Research Group, Faculty of Medicine and Pharmacy, Vrije Universiteit Brussel, Laarbeeklaan 103, 1090 Jette, Brussels, Belgium; ^2^Faculty of Health Care, University College Odisee, Aalst, Belgium; ^3^Department of Work and Organization Studies, KU Leuven, Brussels, Belgium; ^4^Faculty of Business and Sustainable Development, University of Seychelles, Mahe, Seychelles; ^5^Department of Psychiatry & Behavioral Sciences, Stanford University School of Medicine, Stanford, California, USA; ^6^SCALab, Lille 3 University & Siric Oncolille, Lille, France

## Abstract

This article reviews the role of the vagus nerve in tumor modulation and cancer prognosis. We present a systematic review of 12 epidemiological studies examining the relationship between heart rate variability, the main vagus nerve index, and prognosis in cancer patients (survival and tumor markers). These studies show that initially high vagal nerve activity predicts better cancer prognosis, and, in some studies, independent of confounders such as cancer stage and treatments. Since the design of the epidemiological studies is correlational, any causal relationship between heart rate variability and cancer prognosis cannot be inferred. However, various semi-experimental cohort studies in humans and experimental studies in animals have examined this causal relationship. The second part of this paper presents a comprehensive review including human and animal cohort and experimental studies showing that vagotomy accelerates tumor growth, while vagal nerve activation improves cancer prognosis. Based on all reviewed studies, it is concluded that the evidence supports a protective role of the vagus nerve in cancer and specifically in the metastatic stage.

## 1. Introduction

Cancer remains the second leading cause of mortality worldwide, with prostate cancer being the most prevalent cancer type in men and breast cancer in women [1]. Cancer is a complex condition since it includes several hundreds of different types, and because it involves and is affected by multiple body systems, despite beginning as uncontrolled proliferation of a group of cells. Nevertheless, several hallmarks characterize most if not all cancers including sustained cell proliferative signalling, evasion of tumor growth suppressors, resisting cell death (or apoptosis), enabling replicative immortality of cells, induction of angiogenesis, and finally performing invasion and migration (metastasis). Two crucial etiological factors which contribute to these six hallmarks are genetic changes or instability and the immune inflammatory response which contributes to all stages of tumorigenesis [2, 3]. Importantly, studies have shown that three basic biological factors contribute to the onset and progression of tumorigenesis, namely, (1) oxidative stress leading to DNA damage (e.g., [4]); (2) inflammation which contributes to escape from apoptosis, angiogenesis, and metastasis [3, 5]; and (3) excessive sympathetic activity, which affects where cancer cells will metastasize [6–8]. Can there be one factor common to these three factors which contribute to cancer, which inhibits all three and which predicts cancer prognosis as well? We propose that the vagus nerve may fulfil all these requirements. Vagus nerve stimulation reduces oxidative stress [9], informs the brain about inflammation [10], and profoundly inhibits inflammation [11], and of course the vagus nerve inhibits sympathetic activity since it is a major branch of the parasympathetic nervous system [12]. A recently discovered new pathway revealed that vagal nerve stimulation increased TFF2, a suppressor of MDSC; thus, vagal nerve stimulation may increase cellular immunity [13]. For these reasons, we hypothesized that vagus nerve activity may have a prognostic and protective role in cancer [14, 15]. This article will review the epidemiological evidence for its protective role in cancer.

## 2. The Vagus Nerve

The vagus nerve, also called the wandering nerve, is the 10^th^ cranial nerve, descending from various sublocations within the brain medulla, descending in the upper neck between the internal jugular vein and the internal carotid artery. The vagus nerve then innervates multiple visceral organs including the heart, pancreas, lungs, and gastrointestinal tract. The vagus nerve is a complex homeostatic system, which operates via multiple neurotransmitters and affects several systems (cardiovascular, neuroendocrine, and immunological) [11]. It can sense peripheral inflammation and transmits action potentials from the periphery to the brain stem. This in turn leads to the generation of action potentials in the descending vagus nerve, where proinflammatory cytokine production is inhibited [16]. The vagus nerve is known for its protective effects in many pathological conditions. The molecular basis of this anti-inflammatory circuit, termed the cholinergic anti-inflammatory pathway, includes the neurotransmitter acetylcholine interacting with the alpha-7 nicotinic acetylcholine receptor subunit expressed on monocytes, macrophages, and other cytokine producing cells [11]. Signal transduction through this receptor inhibits cytokine release, suppresses inflammation, and has a protective role in many conditions [15]. Many studies have shown the importance and protective effects of the vagus nerve in importance diseases such as cardiovascular disease, Alzheimer's disease, and autoimmune disease [17–22]. On the other hand, low vagus nerve activity has been related to poor outcome, and vagus nerve stimulation with a good outcome in other conditions such as irritable bowel syndrome, metabolic syndrome, diabetes, sepsis, pancreatitis, depression, pain, and epilepsy [23–29].

Its activity can be measured in a noninvasive manner via the measurement of variability of interbeat cardiac intervals, called heart rate variability (HRV). Indeed, HRV is strongly correlated with actual vagal nerve activity (r = 0.88; 30). Importantly, this nerve has a major homeostatic role: People with high HRV were found to recover physiologically to stress more rapidly on three physiological systems, namely, cardiac, hormonal, and immune, compared to those with lower HRV [30]. In addition, in people with high, but not low HRV, synchronization between brain activity and peripheral immunity in regions that modulate homeostasis has been observed [31]. Thus, the vagus has a crucial communicative and moderating homeostatic role in multiple bodily systems, and its index, HRV, has prognostic roles in various health conditions. Is this the case in cancer as well?

## 3. Purpose of This Systematic Review

Zhou and colleagues [32] recently examined the association between HRV and survival in cancer. They identified a total sample of 1286 patients over six studies. Overall, HRV significantly predicted a reduced risk of death from various cancers (HR = 0. 70; 95% confidence interval: 0.60-0.82, p < 0.001 [32]). However, they did not include studies, which also used other clinical outcomes such as tumor markers, enabling more comprehensive examination of the prognostic role of HRV in cancer. Furthermore, they excluded studies with terminal cancer patients, but such information is also important to point at the prognostic role of HRV in the full spectrum of cancer stages and severity. Also, no evaluation of the studies' quality was performed, which can enable one to test the HRV-prognosis relationship in the methodologically better studies only and to inform future studies in a systematic manner how to improve scientifically. Furthermore, no experimental studies were discussed. This review aimed to address these gaps. Since we hypothesize that the vagus nerve may inhibit three factors that are crucial oncogenic mechanisms (oxidative stress, inflammation, and excessive sympathetic activity), we expect high vagus nerve activity to be predictive of a good prognosis in cancer and to slow down tumor progression. This article systematically reviews the studies, which examined the relationship between HRV and cancer prognosis (survival and tumor markers; epidemiological evidence), followed by experimental studies testing the effects of vagotomy and vagal nerve stimulation on cancer prognosis (experimental evidence).

## 4. Evidence Reviewed

Studies were found by using the following key words: heart rate variability; autonomic nerve system; cancer; prognosis; survival; and tumor marker. We also found studies via references of other studies. The search was performed on Pubmed and the years were not restricted.

The inclusion criteria were as follows: studies were included if they measured HRV, in patients with cancer, included as clinical outcome a tumor marker specific for the cancer sampled in the study or survival, being prospective or historical prospective. Studies using a cross-sectional or case-control design were excluded.

## 5. Methodological Evaluation of Studies

We also evaluated the methodological quality of studies, in order to identify possible weaknesses to point out for future studies, given the potential clinical implications of this line of research. Each study's methodological quality was evaluated, while considering the following issues and ratings: (1) Was HRV measured over at least 5min? (No, Yes), (2) Was the design prospective (yes) or historical prospective (No)? (3) Were patients with cardiac diseases or medication excluded, or was this variable statistically controlled or tested for? (No, Yes), (4) Were relevant confounders (e, g., cancer stage, treatment, age) statistically adjusted for in the analysis? (No, Yes), (5) Were effects of HRV tested separately in each cancer type If a study used various types, or was this variable controlled for statistically or methodologically by including one cancer type alone (No, Yes)?

Search History in Pubmed:

1. Using the words HRV heart cancer survival, 17 studies were found, of whom De Couck 2016 [33]; Kim 2015 [34]; Giese-Davis 2015 [35]; Wang 2013 [36] De Couck 2013 [37]; Chiang 2013 [38]; Kim 2010 [39]; and Fadul 2010 [40] were eligible.
2. Using the words HRV heart cancer prognosis, 14 studies were found: 1 new one: Mouton 2012 [41].
3. Using the words “heart rate variability” cancer prognosis, 26 studies were found, of whom only this one was new and eligible: Hoffmann 2001 [42].
4. Using the words “heart rate variability” cancer survival, 35 studies were found, of whom these new and eligible studies were found: Guo 2015 [43]; Chiang 2010 [44]. Another study presented data of samples used in previous studies and tested whether HRV moderates the effects of cancer stage on tumor markers [45]. Since it was a reanalysis of other studies reported here, it was not included in this review. These 12 studies constituted the sample of studies for the epidemiological evidence of this review study (see Table 1).

## 6. Studies of HRV and Cancer

### 6.1. HRV and Cancer Survival

To date, 12 studies have investigated the association between vagal nerve activity and prediction of prognosis in cancer with a total sample of 1822 patients. Hoffmann et al. [42], Chiang et al. [44], and Fadul et al. [40] found that heart rate variability (HRV) predicts survival time. The emerging evidence is quite consistent and demonstrates a prognostic role of vagal activity. We shall now describe each study in detail.

More specifically,* Hoffmann and colleagues [42]* demonstrated a significantly higher mortality for patients with carcinoid heart disease combined with reduced HRV, compared to patients without carcinoid heart disease who also had normal HRV (p=0.04).* Chiang and colleagues [44]* showed a significant correlation between survival time and HF-HRV (r=0.44, p=0.01) in terminal hepatocellular carcinoma patients. However, these three studies did not statistically control for the effects of important confounders such as treatment, cancer stage, gender, and age, and some even included cardiac patients, which could have influenced the HRV parameters themselves.* Fadul and colleagues [40]* found a trend towards a significant association between overall survival and SDNN (p=0.056) in advanced cancer. No significant associations were found between survival and the frequency domain measures (p>0.05). Furthermore, the adjusted hazard ratio for death in patients with abnormal HRV was 6.4 compared with a normal HRV, reflecting indeed a large effect size.* Kim et al. [39]* on the other hand demonstrated that terminal cancer patients with high SDNN survived significantly longer than those with low SDNN, independent of confounders. However, while they statistically controlled for confounders, they included several types of cancer and did not statistically adjust for cancer type.* Chiang et al. [38], Wang et al. [36], De Couck et al. [33],* and* Kim et al. [34]* also showed that HRV predicted survival, and these relationships were mostly independent of important confounders. In the study by* Chiang et al. [38]*, the natural log transformation of HF-HRV (high frequency heart rate variability) below 2 was a significant predictor of risk of surviving 7 days or less compared to patients with a higher HF-HRV.* De Couck et al. [33]* on the other hand found that SDNN significantly predicted survival in patients with advanced pancreatic cancer, and that the SDNN-survival relationship was statistically mediated by reduced levels of C-reactive protein (CRP). This study was the only one which tried to reveal the possible mechanism of the role of the vagus nerve in cancer prognosis. Finally, they also found that, in patients surviving longer than one month, HRV showed the expected inverse correlation with CRP, while in patients surviving less than one month, HRV was unrelated to CRP. This suggests that in patients with neuroimmunomodulation (an inverse HRV-CRP relationship), survival may be longer, even in a severe cancer such as advanced pancreatic cancer. These findings are in line with their hypothesized model where the vagus nerve may modulate cancer progression by inhibiting inflammation [14]. At last,* Wang et al. [36]* found that SDNN predicted survival, independent of confounders, and more specifically, that in patients with SDNN ≥ 10msec, the median survival time was 8 months, compared to a median survival time of 1.8 months in patients with SDNN < 10msec. Finally, in the study by* Kim et al. [34],* SDNN significantly predicted poor survival by univariate analysis, though not multivariate analyses (e.g., age, gender, performance status, and stage).* De Couck et al. [37]* found in nonsmall cell lung cancer patients no significant correlation between SDNN or RMSSD and overall survival nor with survival time. However, in a further exploratory analysis, in the group below age 65, SDNN and RMSSD significantly predicted survival time, independent of confounders (r=0.278, p = .032; r=0.282, p=0.029, respectively), but not in people over 65. This shows that, in some cancers, perhaps in one where the HRV is obviously adversely affected by the disease and age such as lung cancer, the prognostic value of HRV may be moderated by age.* Giese-Davis et al. [35]* reanalyzed data from an existing cohort of women with metastatic and recurrent breast cancer. Using the vagal nerve index of high frequency-HRV (HF-HRV), they found that, in the full sample (N = 87), higher HV-HRV significantly predicted longer survival in a long-term follow-up. Furthermore, this result was then found to be only significant in women without visceral metastases. Finally, they found that the predictive validity of HF-HRV improved when dividing it by patients' heart rate (HR), thus reflecting a more vagal/sympathetic ratio. Though attention was given to confounders, no full multiple regression analysis, controlling for all relevant confounders, in one analysis was performed. Finally,* Guo et al. [43]* performed a historical prospective study and examined in n = 651 cancer patients their HRV (measured during 20-24 hours) and its relationship with survival. They used a cut-off of SDNN = 70msec. Interestingly, the group of patients with SDNN < 70msec included more men, more patients with hematological malignancies, and patients consuming antidepressants from the SSRI family. Looking at the follow-up time when 25% of the sample had died, in those with low SDNN this occurred after 18.7 weeks, while in those with higher SDNN this occurred at week 78.9. Finally, SDNN was a significant predictor of survival, independent of age, cancer stage, and performance status. However, the investigators did not statistically control for cancer type, which could affect both HRV [46] and survival. Nevertheless, this study informs us the HRV predicts survival in a heterogeneous sample of cancer, and low HRV may also be associated with more prevalence of hematological cancers.

### 6.2. HRV and Tumor Markers

*Mouton et al. [41]* and* De Couck et al. [37]* extended these results to the prediction of tumor burden, using serum levels of tumor markers as outcome, while considering multiple confounders. While such markers are not akin to survival, they are used by clinicians to indicate response to treatment and do predict survival in many cancers [47].

*Mouton et al. [41]* demonstrated in a multivariate partial correlation that SDNN was a significant predictor of carcinoembryonic antigen (CEA) levels at 1 year from diagnosis (r=-0.417, p=0.007) in patients with colon cancer. However, when splitting the sample into palliative versus curative, the HRV-CEA relation occurred only in the patients receiving palliative treatment (r=-0.58, p=0.018). Furthermore, patients with low SDNN (<20ms) had significantly higher CEA at 1 year (p=0.006) and even at study entry than patients with higher initial SDNN.* De Couck et al. [37]* showed that SDNN and RMSSD were significant predictors of prostate-specific antigen (PSA) levels at 6 months in prostate cancer patients, controlling for numerous confounders (r=-0.434, p=0.004; r=-0.437, p=0.004, respectively). RMSSD was also found to be a significant predictor of PSA levels at 2 years (r=-0.381, p=0.0125). Furthermore, this was particularly significant in patients with metastatic prostate cancer (r=-0.895, p<0.05), indicating moderation by stage.

## 7. Summary of Study Findings

The 12 studies reviewed above show quite a consistent picture: HRV has prognostic value in cancer, predicting both survival and tumor markers, in several cancers. However, several studies lacked sufficient sample sizes, some studies did not statistically control for important confounders such as cancer stage, treatment, or cancer type, some used too brief measures of HRV, and finally several studies used a historical prospective design. These results are in line with the meta-analysis of Zhou et al. [32], which included only six studies. However, the present review includes double the number of studies and extends these results to predicting tumor markers as well. Importantly, some studies' results also suggest that the predictive value of HRV may especially be strong in advanced stages of cancer [32, 33].

Keeping in mind these limitations, the results of most of the reviewed studies demonstrate that higher initial vagus nerve activity predicts a better cancer prognosis. The relative consistency in the studies reviewed above, across samples with different types of cancer and stages and types of HRV measures, point to a robust prognostic role vagal nerve activity has in cancer. Furthermore, even if we only include the studies that statistically adjusted for confounders and hence have better methodology, 100% of these studies reached the same conclusion. It also seems consistent that there is a significant positive association between HRV and better prognosis, mainly in advanced stages. It is possible that, in earlier tumor stages, treatments such as chemotherapy and radiotherapy are more successful in reducing tumor size and in impacting tumor markers. Such strong therapeutic effects may leave less of a margin for vagal nerve activity to influence the process. In contrast, these treatments may have less impact in later, advanced stages, while (systemic) vagal activity could possibly be of more importance in affecting prognosis. It is also possible that the three factors inhibited by the vagus nerve (oxidative stress, inflammation, and sympathetic activity) may play a more important role in advanced cancer stages, thus possibly increasing the prognostic role of the vagus in later stages. Regardless of the mechanisms, the studies reviewed here call for seriously considering to add HRV to the clinical estimation of prognosis in oncology as well, given its consistent and independent role found in the 12 studies reviewed above. This is crucial because until now, many of the prognostic factors are composed of clinical symptoms and signs, as well as clinician estimates. However, these are affected by physicians' clinical experience. Hence, addition of noninvasive and objective HRV measurements for estimating patients' prognosis could overcome these issues.

Concerning the evaluation of the studies' methodological quality, the mean (SD) score was 4.00 (1.13) and the range was between 2 and 6. The maximal possible evaluation score was 6. If we only consider the studies with ≥4 out of 6, 9 out of 12 studies (75%) had adequate-high methodology. Of these methodologically better studies, in all (100%), HRV significantly predicted either tumor marker levels of patient survival at follow-up.

## 8. Cohort and Experimental Evidence: Effects of Vagotomy on Cancer

Since the design of the epidemiological studies was correlational, we cannot infer any causal relationships between HRV and cancer prognosis. Vagal nerve activity may also be affected by cancer [46]. However, various semiexperimental cohort studies in humans and experimental studies done in animals have examined the relationship between vagotomy and cancer prognosis, with the animal studies enabling one to infer causality. Vagotomy is a surgical sectioning of fibers of the vagus nerve, previously used to diminish acid secretion in the stomach and control a duodenal ulcer [48]. This is an irreversible procedure, whereas a temporary chemical denervation of the vagus can be achieved by administering capsaicin [49]. The latter specifically activates or destroys small diameter sensory neurons containing the capsaicin receptor.

It is well established that in follow-up studies of vagotomised ulcer patients (mostly vagotomy with drainage or antrectomy), an increased risk of colorectal cancer [50, 51], prostate carcinoma [52], and stomach cancer [51, 53] has been found, as well as increased mortality from pulmonary carcinoma [51, 54], cerebrovascular accidents, and bronchopneumonia [51]. Furthermore, in a population-based cohort study in Sweden, the ratio of observed to expected cases of lung cancer was 2.20 (95% confidence interval = 1.82 to 2.63), with an increase in the ratio with time since the operation. However, among the patients with peptic ulcer with vagotomy, the ratio of observed to expected cases was 1.56 (1.49 to 3.67). These findings show that vagotomy increased the risk of cancer, beyond that attributed to peptic ulcer alone [55]. Vagotomy has quite consistently been shown to enhance experimental carcinogenesis in the stomach in various animal species [56–63]. However, in some human studies, no significant increased or decreased risk of gastric cancer was found in patients vagotomised for benign gastric and duodenal disease [64]. Similarly, some experimental studies in animals could not find an increased risk of cancer as well [65–67].

Following these cohort and experimental vagotomy studies, the group of Erin [68] recently conducted an experimental study in mice bearing the 4T1 mammary carcinoma. One week after receiving a high-dose of capsaicin, female adult BALB/c mice were injected orthotopically with syngeneic 4T1 mammary carcinoma cells. A dose-dependent increase in number and size of metastases to the lungs was observed as a function of capsaicin. However, the primary tumor growth was unaffected. These results are also in line with results in humans where HRV predicts prognosis in advanced cancer stages (see above). A subsequent study by the same group [69] investigated the effects of unilateral mid-cervical vagotomy on the metastases of 4THMpc breast carcinoma cells, injected orthotopically, a week after the vagotomy. Similar results were found: unilateral vagotomy increased visceral metastases to the lung, liver, and kidney, without affecting the growth rate of the primary tumor. These two studies propose than an intact vagus nerve may reduce the number and size of visceral metastases in the context of cancer. Since these were experimental studies, they propose a causal relationship between vagal nerve activity and reduced metastasis of an existing cancer.

Novotny et al. [70] have challenged these results and found opposite results when focusing on nonneuronal cholinergic signals. Nonneuronal acetylcholine (Ach) plays a role in cellular proliferation, differentiation, and migration (e.g., [71]), thus of high relevance to cancer. In a study of human colon cancer tissue, they found higher levels of the acetylcholine (ACh) precursor cholineacetyltransferace (ChAT), higher levels of the Ach inhibitor Acetylcholine esterase (AchE) and higher levels of the alpha 7 nicotinic Ach receptor in tumor tissues than control tissues from the same people [70]. Because this was a cross-sectional study, no inferences can be made about the direction of association or causality. Nevertheless, the results suggest involvement of the cholinergic system in colon cancer development. But in an in vitro study, Pettersson et al. [72] found the presence of ChAT and AchE in the human colon cancer cell line HT-29. Importantly, they found that an inhibitor of the Ach precursor ChAT reduced cancer cell proliferation. These results provide evidence for a causal autocrine and paracrine role of Ach in colon cancer cells. It is possible that, at the in situ level, the cholinergic system promotes some tumor types, while in contrast, the vagus nerve at the systemic level may slow tumorigenesis. These issues and the conditions in which they occur require future serious investigation.

## 9. Effects of Vagus Nerve Stimulation on Cancer Progression

The previous studies have demonstrated an association and experimental causal relationships between impaired vagus nerve activity and onset or worse prognosis in cancer. These studies support the importance of an intact vagus nerve in ‘protecting' against a poor cancer prognosis. The next question of course is whether vagus nerve activation has therapeutic effects in cancer, which has clear clinical implications for cancer therapy. The vagus nerve can be stimulated in different ways. An implanted human vagus nerve stimulation (VNS) device has been FDA approved for refractory epilepsy for more than 10 years [73] and is undergoing clinical trials for resistant depression [74]. Another form of an implanted VNS, operating by stimulating baroreceptors, was found to increase HRV parameters as well, in hypertensive patients [75]. Direct electrical stimulation of the vagus nerve attenuates TNF*α*-production during experimental models of endotoxaemia, haemorrhagic shock, and other conditions of cytokine excess [18, 25, 76–78]. However, the voltage and frequency of the stimulation required to activate the cholinergic anti-inflammatory pathway are below the threshold required to activate cardiac vagal fibers, and so no significant effects on HR or HRV have been observed [79]. Furthermore, some noninvasive techniques to stimulate the vagus nerve exist as well. Transcutaneous vagus nerve stimulation (t-VNS) has been shown to attenuate levels of the inflammatory mediator high mobility group box 1 (HMGB1) and improve survival in a murine sepsis model [25]. In a recent study, a new t-VNS device was also found to reduce various inflammatory markers (interleukin 1 beta, interleukin-8, TNF, monocyte chemoattractant protein 1, and macrophage inflammatory protein 1 alpha) in humans [80]. t-VNS was also found to reduce depression by 50%, in two recent human trials [81].

Studies have also demonstrated that multiple forms of meditation can alter the parasympathetic component of HRV and can have a positive effect on cardiac autonomic tone [82–84]. Similarly, relaxation therapy and HRV-biofeedback, both behavioral methods, can significantly increase the parasympathetic component of HRV, reflecting an increase in vagus nerve activity [85–89]. Furthermore, some therapeutic agents targeting the cholinergic anti-inflammatory pathway through action on the vagus nerve have been developed. Examples are *α*7nAChR agonists, the anti-inflammatory compound GTS-21, or CNI-1493, also called Semapimod, which is an anti-inflammatory drug working via an intact vagus nerve. The intact CNS-vagus nerve pathway is required for the anti-inflammatory effects, since surgical vagotomy abrogates the anti-inflammatory effects of Semapimod [76, 77]. Furthermore, an increased efferent vagus nerve activity has been observed after administration of Semapimod, demonstrating its vagal activating potential [90].

Only two studies investigated the effects of Semapimod and one pilot study the effects of HRV-biofeedback directly on cancer. Kemeny et al. [91] investigated the effects of CNI-1493 in combination with IL-2 on a hepatoma tumor. The use of IL-2 as an antineoplastic agent has been limited by the serious toxicities accompanied by the doses required to fight the tumor. When CNI-1493 was administered in conjunction with continuous IL-2 to animals with preexisting tumors, a 10-fold higher dose of IL-2 could be infused, and all animals had a tumor response. Thus, CNI-1493 did not interfere with the antitumoral activity of IL-2 but reduced its associated toxicity. Interestingly, when they compared the tumor volumes in the control group versus the group who received CNI-1493, a smaller tumor volume was found in the latter (though they did not statistically test this). A phase I study in cancer patients demonstrated the safety of the compound CNI-1493 and confirmed its activity in inhibiting TNF synthesis in humans [92].

Indeed, a more recent study conducted by Erin et al. [93] examined the antitumoral effects of vagus nerve activation by Semapimod in mice. Balb/c mice were injected with CNI-1493 (4mg/kg) two days after orthotopic inoculation of 4THM breast carcinoma cells. When measuring the tumor weight approximately 25-28 days after injection of the cells, the tumor weight was significantly decreased in the Semapimod-injected animals. Furthermore, the Semapimod animal group had significantly less macroscopic lung and liver metastases compared to the control group (p<0.05).

Interestingly, a recent matched-controlled pilot study of our group showed positive effects of HRV-biofeedback (HRV-B) in metastatic colon cancer patients. The patients (N = 3) performed daily 20min of HRV-biofeedback for three months, in addition to receiving chemotherapy. They were each retroactively matched to a control patient (N = 3 in total) with the same cancer, same stage, same line of chemotherapy, and similar levels of the tumor marker carcinoembryonic antigen (CEA) at baseline. While in controls, CEA levels hardly changed, patients performing HRV-B showed a clear sharp decline in CEA levels, which by three months tended to be significantly lower than in controls (p < 0.06).

Thus, various forms for vagus nerve activation are available, and their effects on tumor growth and patients' prognosis require careful testing in future studies.

## 10. Novel Scientific Insights

### 10.1. Vagal Influences at the Metastatic Stage

The above studies showed that vagus nerve activity is most strongly correlated with cancer prognosis in metastatic cancer patients. Not only the correlational studies support this, but also the experimental studies of Erin et al. [68, 69] in which they showed that vagal denervation had most influence on the metastases but not on the primary tumor. How can this be explained?

It is possible that, in earlier tumor stages, treatments such as chemotherapy and radiotherapy are more successful in reducing tumor size and in impacting tumor markers. Such strong therapeutic effects may leave less of a margin for vagal nerve activity to influence the process. In contrast, these treatments may have less impact in later, advanced stages, while (systemic) vagal activity could possibly be of more importance in affecting prognosis. In addition, during the metastatic stage, all three mechanisms thought to underlie the effects of the vagus on tumors, namely, inflammation, oxidative stress, and sympathetic activation [3, 5, 6, 94], may have a greater role in prognosis. This would then potentially enable us to observe greater impact of the vagus nerve on these three processes and on prognosis in the advanced stages of cancer specifically.

The study by Magnon et al. [95] showed that the sympathetic fibers were important at the early stages of tumorigenesis, while the parasympathetic fibers were affecting tumor progression at the later metastatic stage, however, in the other direction of our hypothesized antitumoral effects. Further research is needed to examine the actual role and mechanisms of the vagus nerve in cancer in general and in the metastatic stage specifically and to reveal when it has antitumor and tumor-promoting effects.

## 11. Systemic versus Local Vagal Effects

Most of the correlational studies testing HRV and cancer prognosis found a protective effect of high vagal activity in cancer and represent a systemic influence. Furthermore, the study by Erin et al. [93], which is experimental, is also performed at the systemic level. In contrasts, most of the studies with acetylcholine found a tumor-promoting effect but represent a more local effect.

Given the mainly homeostatic role of this nerve in relation to multiple physiological systems [30], the vagus nerve's effects at the systemic level may slow down tumorigenesis, while the nerve's primary neurotransmitter acetylcholine at the local level may promote tumors. Similar dual effects can be observed with corticotrophic releasing hormone (CRH), where it has an anti-inflammatory effect when originating from the brain, but it is proinflammatory when released locally from nerve endings at sites of inflammation [96]. A similar dual action can be seen with norepinephrine and other corticosteroids. The study of Kerzerho et al. [97] demonstrated that local administration of corticosteroids had no effect on the development of immune/mucosal tolerance in contrast to systemically applied. Furthermore, not the concentration, but the route of administration of corticosteroids affected the immune outcome.

Furthermore, since 80% of the vagal fibers are afferent and transmit information to the brain (e.g., influence the HPA axis), there is a greater chance for the vagus to influence systemic, rather than local and in situ functions. Thayer et al. [98] contend in a meta-analysis that dynamic connections between the amygdala and medial prefrontal cortex, which evaluate threat and safety, help regulate HRV through their connections with the nucleus tractus solitarius (NTS). They propose that vagally mediated HRV is linked to higher-level executive functions. Furthermore, HRV reflects the functional capacity of these brain structures that support working memory and emotional and physiological self-regulation. Interestingly, one region, namely, the anterior cingulate cortex, seems to be related to both vagal nerve activity [99] and to cellular anticancer immunity (NK cells) [100]. At last, the efferent vagus nerve may also have a systemic effect, via inhibiting cytokine production in macrophages residing in the spleen (via a mechanism that is not yet sufficiently understood) [101]. All these factors may need to be considered in future studies. This is why it is important to test this topic in both levels and that the conclusions of one level (systemic) may not be the same for the other level (local). Thus, the vagus nerve could affect cancer via afferent-central neuroimmunomodulation as well.

## 12. Conclusions

This systematic review examined the evidence linking vagus nerve activity near diagnosis, as indexed by HRV, and prognosis in cancer patients. By evaluating the methodological quality of the identified studies, we were also able to check this issue in the methodologically better studies. It is concluded that a great majority of studies showed that HRV significantly predicts either tumor marker levels of patients' vital status, at various follow-ups in different cancers. Importantly, 100% of the methodologically better studies found the same result. Thus, we conclude that a relatively high vagus nerve activity predicts better prognosis in several cancers, independent of important prognostic factors such as age, cancer stage, or treatments. The experimental evidence from animal research confirms this conclusion and provides evidence for a causal relationship between an intact vagus nerve and slower tumor progression (e.g., [54, 55]) or between vagal activation via Semapimod, and less metastasis [79].

Some new insights came to light. First of all, the studies seem to point at a strong influence of vagal nerve activity specifically in the metastatic stage. It is hypothesized that during this stage, the three mechanisms, namely, inflammation, oxidative stress, and sympathetic activation [3, 5, 6, 81], which are etiological to cancer prognosis, may have a greater role. This would then potentially enable us to observe greater impact of the vagus nerve on these three processes, and on prognosis in the advanced stages of cancer specifically.

Secondly, and on the other hand, some studies find the opposite pattern when examining the effects of the vagal neurotransmitter, Ach, and its related parameters, and tumorigenesis [56]. Most of the studies with acetylcholine found a tumor-promoting effect but represent a more local effect. It is possible that systemic effects of the vagus may have antitumoral effects, while nonneuronal Ach has tumorigenic effects in some cancers.

Future studies need to improve various methodological shortcomings identified in the studies reviewed here. First, HRV measures of at least 5 minutes are needed, to increase the reliability of these measures. Second, it is possible that the prognostic value of HRV may be even stronger if measured also during one minute of deep paced breathing, which is expected to increase HRV. This would examine whether vagal reactivity predicts cancer prognosis. Importantly, all studies should statistically control for known confounders, when examining any new prognostic estimator. Finally, studies need to examine the mechanism. Does HRV predict cancer prognosis via reducing inflammation or by increasing anticancer immunity, by reducing oxidative stress or by reducing excessive sympathetic activity [15]?

## Figures and Tables

**Table 1:** Summary of available studies of HRV and cancer. SDNN=standard deviation of normal to normal R-R intervals).

**Study**	**Design**	**Sample size**	**Cancer type**	**ECG duration**	**Controlled for confounders**	**Cardiac patients included?**	**Results SDNN **	**QOL ** **1-6**
Hoffmann 2001	Prospective	35, both genders	Metastatic Carcinoid syndrome (CS)	24-hour Holter	No	No	SDNN > 100 + CS Predicted mortality	3

Kim 2010	Prospective	68, both genders	All types of primary tumours	5 minute	Yes, full	No	SDNN predicted Duration of survival	4

Fadul 2010	Historical Prospective	47, males	All types of advanced disease (lung and gastro)	20 minute	No	Yes	SDNN tended to predict survival	2

Chiang 2010	Prospective	33, both genders	Terminal Hepatocellular carcinoma	5 minute	No	Yes	HF-HRV correlate with time-till death	4

Mouton 2012	Historical Prospective	38, both genders	Colorectal cancer	10 seconds	Yes, full	No	SDNN predicted levels of CEA over 12 monts	4

De Couck 2013	Historical Prospective	113, male 133, both genders	Prostate cancer Non-small cell lung cancer	10 seconds	Yes, full	No	SDNN predicted PSA and survival time in lung cancer in < 65 years	4

Chiang 2013	Prospective	138, both genders	Liver, colon, stomach, head & neck, pancreatic, genitourinary, oesophagal	5min	Yes, partial	Yes	HF-HRV predicted risk of survival < 7 days	4

Wang 2013	Prospective	40, both genders	Lung, breast & others with brain metastases	5min	Yes, full	No	SDNN predicted survival	6

De Couck 2016	Historical Prospective	272, both genders	Advanced pancreatic cancer	10 seconds	Yes, full	Yes, but this variable was controlled for	High SDNN Predicted Double survival	4

Kim 2015	Prospective	167, both genders	Advanced non-small-cell lung cancer				SDNN predicted survival	6

Giese-Davis 2015	Prospective	87, only women	Metastatic-recurrent breast cancer	ECG 5min	Yes, partial	Yes	SDNN and SDNN/HR predicted survival	3

Guo 2015	Historical prospective	651, both genders	Several cancers	ECG, 20-24 hours	Yes, partial		SDNN < 70ms predicted shorter survival	4

## References

[1] Global Burden of Disease Cancer Collaboration (2017). Global, regional, and national cancer incidence, mortality, years of life lost, years lived with disability, and disability-adjusted life years for 32 cancer groups, 1990 to 2015: a systematic analysis for the Global Burden of Disease Study 2015. *JAMA Oncol*.

[2] Hanahan D., Weinberg R. A. (2011). Hallmarks of cancer: the next generation. *Cell*.

[3] Voronov E., Shouval D. S., Krelin Y. (2003). IL-1 is required for tumor invasiveness and angiogenesis. *Proceedings of the National Acadamy of Sciences of the United States of America*.

[4] Valko M., Izakovic M., Mazur M., Rhodes C. J., Telser J. (2004). Role of oxygen radicals in DNA damage and cancer incidence. *Molecular and Cellular Biochemistry*.

[5] Mantovani A., Allavena P., Sica A., Balkwill F. (2008). Cancer-related inflammation. *Nature*.

[6] Entschladen F., Drell VI T. L., Lang K., Joseph J., Zaenker K. S. (2004). Tumour-cell migration, invasion, and metastasis: Navigation by neurotransmitters. *The Lancet Oncology*.

[7] Thaker P. H., Han L. Y., Kamat A. A. (2006). Chronic stress promotes tumor growth and angiogenesis in a mouse model of ovarian carcinoma. *Nature Medicine*.

[8] Thaker P. H., Sood A. K., Ramondetta L. M. (2013). Importance of adrenergic pathways in women's cancers. *Cancer Biomarkers*.

[9] Tsutsumi T., Ide T., Yamato M. (2008). Modulation of the myocardial redox state by vagal nerve stimulation after experimental myocardial infarction. *Cardiovascular Research*.

[10] Ek M., Kurosawa M., Lundeberg T., Ericsson A. (1998). Activation of vagal afferents after intravenous injection of interleukin-1*β*: Role of endogenous prostaglandins. *The Journal of Neuroscience*.

[11] Tracey K. J. (2009). Reflex control of immunity. *Nature Reviews Immunology*.

[12] Maki A., Kono H., Gupta M. (2007). Predictive power of biomarkers of oxidative stress and inflammation in patients with hepatitis C virus-associated hepatocellular carcinoma. *Annals of Surgical Oncology*.

[13] Dubeykovskaya Z., Si Y., Chen X. (2016). Neural innervation stimulates splenic TFF2 to arrest myeloid cell expansion and cancer. *Nature Communications*.

[14] Gidron Y., Perry H., Glennie M. (2005). Does the vagus nerve inform the brain about preclinical tumours and modulate them?. *The Lancet Oncology*.

[15] De Couck M., Mravec B., Gidron Y. (2012). You may need the vagus nerve to understand pathophysiology and to treat diseases. *Clinical Science*.

[16] Tracey K. J. (2002). The inflammatory reflex. *Nature*.

[17] Guarini S., Cainazzo M. M., Giuliani D. (2004). Adrenocorticotropin reverses hemorrhagic shock in anesthetized rats through the rapid activation of a vagal anti-inflammatory pathway. *Cardiovascular Research*.

[18] Guarini S., Altavilla D., Cainazzo M.-M. (2003). Efferent vagal fibre stimulation blunts nuclear factor-*κ*B activation and protects against hypovolemic hemorrhagic shock. *Circulation*.

[19] Ottani A., Giuliani D., Galantucci M. (2010). Melanocortins counteract inflammatory and apoptotic responses to prolonged myocardial ischemia/reperfusion through a vagus nerve-mediated mechanism. *European Journal of Pharmacology*.

[20] Vonck K., Raedt R., Naulaerts J. (2014). Vagus nerve stimulation. . .25 years later! What do we know about the effects on cognition?. *Neuroscience & Biobehavioral Reviews*.

[21] Li J., Xie H., Wen T., Liu H., Zhu W., Chen X. (2010). Expression of high mobility group box chromosomal protein 1 and its modulating effects on downstream cytokines in systemic lupus erythematosus. *The Journal of Rheumatology*.

[22] Feldmann M., Brennan F. M., Maini R. N. (1996). Role of cytokines in rheumatoid arthritis. *Annual Review of Immunology*.

[23] Bonaz B., Bazin T., Pellissier S. (2018). The Vagus Nerve at the Interface of the Microbiota-Gut-Brain Axis. *Frontiers in Neuroscience*.

[24] Pal G. K., Adithan C., Ananthanarayanan P. H. (2013). Sympathovagal Imbalance contributes to prehypertension status and cardiovascular risks attributed by insulin resistance, inflammation, dyslipidemia and oxidative stress in first degree relatives of type 2 diabetics. *PLoS ONE*.

[25] Huston J. M., Gallowitsch-Puerta M., Ochani M. (2007). Transcutaneous vagus nerve stimulation reduces serum high mobility group box 1 levels and improves survival in murine sepsis. *Critical Care Medicine*.

[26] van Westerloo D. J., Giebelen I. A., Florquin S. (2006). The Vagus Nerve and Nicotinic Receptors Modulate Experimental Pancreatitis Severity in Mice. *Gastroenterology*.

[27] Müller H. H., Moeller S., Lücke C., Lam A. P., Braun N., Philipsen A. (2018). Vagus Nerve Stimulation (VNS) and Other Augmentation Strategies for Therapy-Resistant Depression (TRD): Review of the Evidence and Clinical Advice for Use. *Frontiers in Neuroscience*.

[28] Chakravarthy K., Chaudhry H., Williams K., Christo P. J. (2015). Review of the Uses of Vagal Nerve Stimulation in Chronic Pain Management. *Current Pain and Headache Reports*.

[29] Morris G. L., Mueller W. M. (1999). Long-term treatment with vagus nerve stimulation in patients with refractory epilepsy. *Neurology*.

[30] Weber C. S., Thayer J. F., Rudat M. (2010). Low vagal tone is associated with impaired post stress recovery of cardiovascular, endocrine, and immune markers. *European Journal of Applied Physiology*.

[31] Ohira H., Matsunaga M., Osumi T. (2013). Vagal nerve activity as a moderator of brain-immune relationships. *Journal of Neuroimmunology*.

[32] Zhou X., Ma Z., Zhang L. (2016). Heart rate variability in the prediction of survival in patients with cancer: A systematic review and meta-analysis. *Journal of Psychosomatic Research*.

[33] Couck M. D., Maréchal R., Moorthamers S., Laethem J.-L. V., Gidron Y. (2016). Vagal nerve activity predicts overall survival in metastatic pancreatic cancer, mediated by inflammation. *Cancer Epidemiology*.

[34] Kim K., Chae J., Lee S. (2015). The role of heart rate variability in advanced non-small-cell lung cancer patients. *Journal of Palliative Care*.

[35] Giese-Davis J., Wilhelm F. H., Tamagawa R. (2015). Higher vagal activity as related to survival in patients with advanced breast cancer: An analysis of autonomic dysregulation. *Psychosomatic Medicine*.

[36] Wang Y.-M., Wu H.-T., Huang E.-Y., Kou Y. R., Hseu S.-S. (2013). Heart rate variability is associated with survival in patients with brain metastasis: A preliminary report. *BioMed Research International*.

[37] Couck M. D., Brummelen D. V., Schallier D., Grève J. D., Gidron Y. (2013). The relationship between vagal nerve activity and clinical outcomes in prostate and non-small cell lung cancer patients. *Oncology Reports*.

[38] Chiang J.-K., Kuo T. B. J., Fu C.-H., Koo M. (2013). Predicting 7-Day Survival Using Heart Rate Variability in Hospice Patients with Non-Lung Cancers. *PLoS ONE*.

[39] Kim D. H., Kim J. A., Choi Y. S., Kim S. H., Lee J. Y., Kim Y. E. (2010). Heart rate variability and length of survival in hospice cancer patients. *Journal of Korean Medical Science*.

[40] Fadul N., Strasser F., Palmer J. L. (2010). The association between autonomic dysfunction and survival in male patients with advanced cancer: a preliminary report. *Journal of Pain and Symptom Management*.

[41] Mouton C., Ronson A., Razavi D. (2012). The relationship between heart rate variability and time-course of carcinoembryonic antigen in colorectal cancer. *Autonomic Neuroscience: Basic and Clinical*.

[42] Hoffmann J., Grimm W., Menz V. (2001). Prognostic value of heart rate variability analysis in patients with carcinoid syndrome. *Digestion*.

[43] Guo Y., Koshy S., Hui D. (2015). Prognostic Value of Heart Rate Variability in Patients with Cancer. *Journal of Clinical Neurophysiology*.

[44] Chiang J.-K., Koo M., Kuo T. B. J., Fu C.-H. (2010). Association Between Cardiovascular Autonomic Functions and Time to Death in Patients With Terminal Hepatocellular Carcinoma. *Journal of Pain and Symptom Management*.

[45] Gidron Y., De Couck M., De Greve J. (2014). If you have an active vagus nerve, cancer stage may no longer be important. *Journal of Biological Regulators and Homeostatic Agents*.

[46] de Couck M., Gidron Y. (2013). Norms of vagus nerve activity, indexed by heart rate variability, in cancer patients. *Cancer Epidemiology*.

[47] Sharma S. (2009). Tumor markers in clinical practice: General principles and guidelines. *Indian Journal of Medical and Paediatric Oncology*.

[48] Ulloa L. (2005). The vagus nerve and the nicotinic anti-inflammatory pathway. *Nature Reviews Drug Discovery*.

[49] Buck S. H., Burks T. F. (1986). The neuropharmacology of capsaicin: review of some recent observations. *Pharmacol Rev*.

[50] Offerhaus G. J. A., Tersmette A. C., Huibregtse K. (1988). Mortality caused by stomach cancer after remote partial gastrectomy for benign conditions: 40 years of follow up of an Amsterdam cohort of 2633 postgastrectomy patients. *Gut*.

[51] Toftgaard C. (1989). Gastric cancer after peptic ulcer surgery: An historic prospective cohort investigation. *Annals of Surgery*.

[52] Åhsberg K., Olsson H., Staël Von Holstein C. (2009). Increased mortality in prostate carcinoma and smoking-related disease after parietal cell vagotomy: A long-term follow-up study. *Scandinavian Journal of Gastroenterology*.

[53] Stockbrugger R. W., Cotton P. B., Eugenides N., Bartholomew B. A., Hill M. J., Walters C. L. (1982). Intragastric nitrites, nitrosamines, and bacterial overgrowth during cimetidine treatment. *Gut*.

[54] Sharma B. K., Santana I. A., Wood E. C. (1984). Intragastric bacterial activity and nitrosation before, during, and after treatment with omeprazole.. *BMJ*.

[55] Ekbom A. (1998). Risk of Cancer in Ulcerative Colitis. *Journal of Gastrointestinal Surgery*.

[56] Morgenstern L. (1968). Vagotomy, Gastroenterostomy and Experimental Gastric Cancer. *JAMA Surgery*.

[57] Kowalewski K. (1973). Relationship between vagotomy, peptic ulcer and gastric adenocarcinoma in rats fed 2,7-diacetylaminofluorene. *Can JSurg*.

[58] Junghanns K., Seufert R., von Gerstenbergk L., Ivankovic S. (1979). Does vagotomy and pyloroplasty change the location of gastrointestinal tumors?. *World Journal of Surgery*.

[59] Fujita M., Takami M., Usugane M., NamDei S., Taguchi T. (1979). Enhancement of gastric carcinogenesis in dogs given n-methyl-N'-nitro-n-nitrosoguanidine following vagotomy. *Cancer Research*.

[60] Mori H., Domellof L., Weisburger J. H., Williams G. M. (1981). Enhancing effect of vagotomy and pyloroplasty on gastro-intestinal carcinogenesis induced by nitrosamide in hamsters. *GANN Japanese Journal of Cancer Research*.

[61] Tatsuta M., Yamamura H., lishi H. (1985). Promotion by Vagotomy of Gastric Carcinogenesis Induced by N-Methyl-N'-nitro-N-nitrosoguanidine in Wistar Rats. *Cancer Research*.

[62] Tatsuta M., Iishi H., Yamamura H., Baba M., Taniguchi H. (1988). Effects of bilateral and unilateral vagotomy on gastric carcinogenesis induced by N‐methyl‐N′‐nitro‐N‐nitrosoguanidine in wistar rats. *International Journal of Cancer*.

[63] Nelson R. L., Briley S., Vaz O. P., Abcarian H. (1992). The effect of vagotomy and pyloroplasty on colorectal tumor induction in the rat. *Journal of Surgical Oncology*.

[64] Lundegardh G., Ekbom A., McLaughlin J. K., Nyren O. (1994). Gastric cancer risk after vagotomy.. *Gut*.

[65] Bayón Lara A. M., Landa García I., Alcalde Escribano J., Rodríguez Dapena S., Ortega Medina L., Balibrea Cantero J. L. (2001). Colonic carcinogenesis in vagotomyzed rats. *Revista Española de Enfermedades Digestivas*.

[66] Schlag P., Weber E., Meister H., Meyer H. (1982). Animal experiments to assess the risk of cancer in the stomach after vagotomy. *Langenbecks Archiv für Chirurgie*.

[67] Rumpf P., Schacht U., Palomba P., Kremer K., Borchard E. (1977). Chemically induced stomach carcinomas in rats following vagotomy and Bilroth II gastrectomy. *Chirurgisches Forum fur experimentelle und klinische Forschung*.

[68] Erin N., Boyer P. J., Bonneau R. H., Clawson G. A., Welch D. R. (2004). Capsaicin-mediated Denervation of Sensory Neurons Promotes Mammary Tumor Metastasis to Lung and Heart. *Anticancer Reseach*.

[69] Erin N., Akdas Barkan G., Harms J. F., Clawson G. A. (2008). Vagotomy enhances experimental metastases of 4THMpc breast cancer cells and alters Substance P level. *Regulatory Peptides*.

[70] Novotny A., Ryberg K., Heiman Ullmark J. (2011). Is acetylcholine a signaling molecule for human colon cancer progression?. *Scandinavian Journal of Gastroenterology*.

[71] Trombino S., Bisio A., Catassi A., Cesario A., Falugi C., Russo P. (2004). Role of the non-neuronal human cholinergic system in lung cancer and mesothelioma: possibility of new therapeutic strategies. *Current Medicinal Chemistry—Anti-Cancer Agents*.

[72] Pettersson A., Nilsson L., Nylund G., Khorram-Manesh A., Nordgren S., Delbro D. S. (2009). Is acetylcholine an autocrine/paracrine growth factor via the nicotinic *α*7-receptor subtype in the human colon cancer cell line HT-29?. *European Journal of Pharmacology*.

[73] Schachter S. C. (2002). Vagus nerve stimulation therapy summary: Five years after FDA approval. *Neurology*.

[74] Corcoran C., Connor T. J., O'Keane V., Garland M. R. (2005). The effects of vagus nerve stimulation on pro- and anti-inflammatory cytokines in humans: A preliminary report. *Neuroimmunomodulation*.

[75] Wustmann K., Kucera J. P., Scheffers I. (2009). Effects of chronic baroreceptor stimulation on the autonomic cardiovascular regulation in patients with drug-resistant arterial hypertension. *Hypertension*.

[76] Bernik T. R., Friedman S. G., Ochani M. (2002). Pharmacological stimulation of the cholinergic antiinflammatory pathway. *The Journal of Experimental Medicine*.

[77] Borovikova L. V., Ivanova S., Zhang M. (2000). Vagus nerve stimulation attenuates the systemic inflammatory response to endotoxin. *Nature*.

[78] Wang H., Yu M., Ochani M. (2003). Nicotinic acetylcholine receptor *α*7 subunit is an essential regulator of inflammation. *Nature*.

[79] Czura C. J., Tracey K. J. (2005). Autonomic neural regulation of immunity. *Journal of Internal Medicine*.

[80] Lerman I., Hauger R., Sorkin L. (2016). Noninvasive Transcutaneous Vagus Nerve Stimulation Decreases Whole Blood Culture-Derived Cytokines and Chemokines: A Randomized, Blinded, Healthy Control Pilot Trial. *Neuromodulation: Technology at the Neural Interface*.

[81] Hein E., Nowak M., Kiess O. (2013). Auricular transcutaneous electrical nerve stimulation in depressed patients: a randomized controlled pilot study. *Journal of Neural Transmission*.

[82] Kubota Y., Sato W., Toichi M. (2001). Frontal midline theta rhythm is correlated with cardiac autonomic activities during the performance of an attention demanding meditation procedure. *Cognitive Brain Research*.

[83] Paul-Labrador M., Polk D., Dwyer J. H. (2006). Effects of a randomized controlled trial of transcendental meditation on components of the metabolic syndrome in subjects with coronary heart disease. *JAMA Internal Medicine*.

[84] Peng C.-K., Henry I. C., Mietus J. E. (2004). Heart rate dynamics during three forms of meditation. *International Journal of Cardiology*.

[85] Cowan M. J., Kogan H., Burr R., Hendershot S., Buchanan L. (1990). Power spectral analysis of heart rate variability after biofeedback training. *Journal of Electrocardiology*.

[86] Nolan R. P., Kamath M. V., Floras J. S. (2005). Heart rate variability biofeedback as a behavioral neurocardiac intervention to enhance vagal heart rate control. *American Heart Journal*.

[87] Sakakibara M., Hayano J., Oikawa L. O., Katsamanis M., Lehrer P. (2013). Heart rate variability biofeedback improves cardiorespiratory resting function during sleep. *Applied Psychophysiology and Biofeedback*.

[88] Terathongkum S., Pickler R. H. (2004). Relationships among heart rate variability, hypertension, and relaxation techniques. *Journal of Vascular Nursing*.

[89] Van Dixhoorn J., White A. (2005). Relaxation therapy for rehabilitation and prevention in ischaemic heart disease: A systematic review and meta-analysis. *European Journal of Preventive Cardiology*.

[90] Pavlov V. A., Ochani M., Gallowitsch-Puerta M. (2006). Central muscarinic cholinergic regulation of the systemic inflammatory response during endotoxemia. *Proceedings of the National Acadamy of Sciences of the United States of America*.

[91] Kemeny M. M., Botchkina G. I., Ochani M., Bianchi M., Urmacher C., Tracey K. J. (1998). The tetravalent guanylhydrazone CNI-1493 blocks the toxic effects of interleukin-2 without diminishing antitumor efficacy. *Proceedings of the National Acadamy of Sciences of the United States of America*.

[92] Atkins M. B., Redman B., Mier J. (2001). A Phase I Study of CNI-1493, an Inhibitor of Cytokine Release, in Combination with High-Dose Interleukin-2 in Patients with Renal Cancer and Melanoma. *Clin Cancer Res*.

[93] Erin N., Duymuş Ö., Öztürk S., Demir N. (2012). Activation of vagus nerve by semapimod alters substance P levels and decreases breast cancer metastasis. *Regulatory Peptides*.

[94] Armaiz-Pena G. N., Allen J. K., Cruz A. (2013). Src activation by adrenoreceptors is a key switch for tumour metastasis. *Nature Communications*.

[95] Magnon C., Hall S. J., Lin J. (2013). Autonomic nerve development contributes to prostate cancer progression. *Science*.

[96] Agelaki S., Tsatsanis C., Gravanis A., Margioris A. N. (2002). Corticotropin-releasing hormone augments proinflammatory cytokine production from macrophages in vitro and in lipopolysaccharide-induced endotoxin shock in mice. *Infection and Immunity*.

[97] Kerzerho J., Wunsch D., Szely N. (2012). Effects of systemic versus local administration of corticosteroids on mucosal tolerance. *The Journal of Immunology*.

[98] Thayer J. F., Åhs F., Fredrikson M., Sollers J. J., Wager T. D. (2012). A meta-analysis of heart rate variability and neuroimaging studies: implications for heart rate variability as a marker of stress and health. *Neuroscience & Biobehavioral Reviews*.

[99] Matthews S. C., Paulus M. P., Simmons A. N., Nelesen R. A., Dimsdale J. E. (2004). Functional subdivisions within anterior cingulate cortex and their relationship to autonomic nervous system function. *NeuroImage*.

[100] Tashiro M., Itoh M., Kubota K. (2001). Relationship between trait anxiety, brain activity and natural killer cell activity in cancer patients: A preliminary pet study. *Psycho-Oncology*.

[101] Rosas-Ballina M., Olofsson P. S., Ochani M. (2011). Acetylcholine-synthesizing T cells relay neural signals in a vagus nerve circuit. *Science*.

